# Revealing Microbial Siderophores: From Genes to Applications

**DOI:** 10.3390/microorganisms14020393

**Published:** 2026-02-06

**Authors:** Jionglin Cai, Yuting Fang, Xia Liu, Mark Owusu Adjei, Ben Fan

**Affiliations:** Co-Innovation Center for Sustainable Forestry in Southern China, Department of Forest Protection, College of Forestry and Grassland, Nanjing Forestry University, Nanjing 210037, China

**Keywords:** applications, biosynthesis, purification and identification, regulatory genes, siderophore, transport

## Abstract

Iron is an essential micronutrient for nearly all microorganisms, yet its bioavailability is severely limited in most environments. To overcome this restriction, microorganisms produce siderophores, high-affinity iron-chelating molecules that play pivotal roles in microbial iron homeostasis, interspecies competition, and host–pathogen interactions. Despite extensive research, current understanding of siderophore biosynthetic and regulatory diversity remains largely limited to specific models, with comprehensive cross-taxonomic frameworks only beginning to emerge. This review systematically integrates recent advances in the genetic and biochemical foundations of microbial siderophore production, focusing on the two major biosynthetic pathways: nonribosomal peptide synthetase (NRPS)-dependent and NRPS-independent synthetase (NIS). We further elaborate on the diverse transport systems in Gram-negative and Gram-positive bacteria, as well as fungi, alongside the iron-responsive regulators (e.g., Fur) and gene clusters that coordinate iron uptake and utilization. Beyond physiological mechanisms, we discuss how these insights inform emerging applications of siderophores across multiple fields: in medicine, enabling “Trojan horse” antimicrobial strategies; in agriculture, enhancing plant iron uptake and serving as biocontrol agents; in environmental remediation, facilitating heavy-metal detoxification; and in biosensing, acting as selective probes for metals and pathogens. By bridging fundamental mechanisms with practical applications, this review aims to provide an integrative perspective for future exploration of microbial iron homeostasis and its biotechnological potential.

## 1. Introduction

Iron is indispensable for all microorganisms. It serves as a key ecological and evolutionary constraint shaping microbial metabolism and competition. This element functions as a cofactor in redox reactions, enzymatic catalysis, and numerous metabolic processes. Although geochemically abundant, environmental iron predominantly exists as insoluble Fe(III), which imposes strong selective pressure for high-affinity acquisition systems. To overcome this limitation, microorganisms produce siderophores—high-affinity iron chelators—to acquire the iron they require. Beyond iron acquisition, siderophores mediate interspecies interactions, structure competitive networks, and contribute to virulence and host colonization in many pathogens [1].

Because of their high metal affinity and molecular specificity, siderophores hold considerable promise for applications in medicine, agriculture, and environmental biotechnology. Recent studies increasingly focus on the sustainable production, engineering, and translational use of siderophores in antimicrobial therapy, crop protection, and metal recovery [2].

Since the term “siderophore” was first introduced over six decades ago [3], research has expanded rapidly, reflecting its central role in microbial physiology. Nevertheless, our understanding of siderophores remains incomplete. Their remarkable structural and functional diversity has resulted in a predominance of species-specific studies focusing on distinct biosynthetic, transport, and regulatory nuances. Consequently, current concepts are largely shaped by a limited number of bacterial model systems, notably *Escherichia coli*, and by studies of catecholate- or hydroxamate-type siderophores. Broader cross-species and integrative analyses that elucidate conserved biosynthetic and regulatory themes remain to be more systematically investigated. In contrast, siderophore systems in fungi, particularly in phytopathogenic and human-pathogenic species, represent a significant area for future exploration and integration.

This review critically synthesizes recent advances in microbial siderophore biosynthesis, transport, and regulation, with a particular focus on genetic mechanisms and emerging applications across bacterial and fungal systems.

## 2. Types and Identification of Siderophores

**Figure 1:**
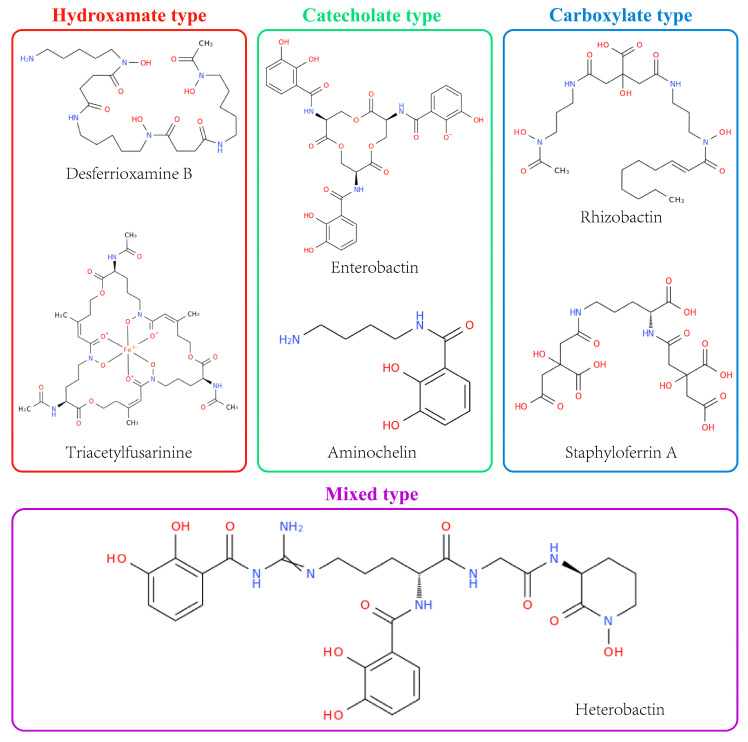
Chemical structures of representative examples of different types of siderophores. Structures adapted from the Chemical Entities of Biological Interest (ChEBI) database.

Over 900 siderophores have been characterized to date [4]. They are classified into four principal types based on their iron-coordinating functional groups: hydroxamates, catecholates, carboxylates, and mixed-type compounds [5] (Figure 1). Hydroxamates coordinate Fe(III) through the oxygen donor atoms of N-hydroxyornithine, catecholates through phenolic hydroxyls, and carboxylates through carboxylate and hydroxyl functionalities; mixed-type siderophores incorporate two or more ligand classes within a single molecule. Their taxonomic distribution reflects species-specific biosynthetic capabilities [6] (Table 1).

At the chemical level, catechol, hydroxamate, and α-hydroxycarboxylate moieties represent the three principal types of bidentate ligands, each conferring high selectivity and affinity for Fe(III). In terms of denticity, hexadentate siderophores, which present six coordinating atoms to Fe(III), typically exhibit the highest thermodynamic stability [7]. The efficiency of chelation is strongly pH-dependent: hydroxamate ligands dominate in acidic soils, whereas catecholate ligands are often more effective under neutral to alkaline environments [8].

Siderophore profiles differ between kingdoms: hydroxamates are predominant in fungi [9], whereas catecholates are predominant in bacteria [10]. Reported Fe(III)–siderophore stability constants vary widely, depending on measurement conditions (e.g., pH, ionic strength, and methodology). This variation reflects the diversity of their ligand chemistry. While high-affinity fungal hydroxamates typically can reach stability constants in the range of 10^29^ to 10^31^, bacterial catecholates exhibit constants as high as 10^50^ [11].

**Table 1:** Main types of siderophores found in different microorganisms.

Name	Types of Siderophores	Producing Microorganism	References
Desferrioxamine B	Hydroxamate	*Streptomyces pilosus*	[12]
Baumannoferrin A	Hydroxamate	*Acinetobacter baumannii*	[13]
Triacetylfusarinine	Hydroxamate	*Aspergillus nidulans*	[14]
Aminochelin	Catecholate	*Azotobacter vinelandii*	[15]
Photobactin	Catecholate	*Photorhabdus luminescens*	[16]
Enterobactin	Catecholate	*Escherichia coli*	[17]
Salmochelin	Catecholate	*Klebsiella pneumoniae*	[18]
Bacillibactin	Catecholate	*Bacillus velezensis*	[19]
Rhizobactin	Carboxylate	*Rhizobium meliloti*	[20]
Staphyloferrin A	Carboxylate	*Staphylococcus aureus*	[21]
Desmalonichrome	Carboxylate	*Fusarium oxysporum*	[22]
Aerobactin	Mixed type	*Escherichia coli*	[23]
Pyoverdine	Mixed type	*Pseudomonas fluorescens*	[24]
Heterobactin	Mixed type	*Rhodococcus erythropolis*	[25]
Ornibactin	Mixed type	*Burkholderia vietnamiensis*	[26]

## 3. Siderophore Biosynthesis in Microorganisms

Siderophore biosynthesis primarily proceeds via two routes: nonribosomal peptide synthetase (NRPS)-dependent and NRPS-independent (NIS) [27]. These two pathways can be described in terms of their biosynthetic steps and the associated gene clusters.

### 3.1. Nonribosomal Peptide Synthetase Pathway

According to the SIDERITE database, about two thirds of known siderophores are synthesized through the NRPS pathway [4]. NRPSs are large multimodule enzymes typically composed of three basic domains—adenylation (A), thiolation (T) and condensation (C)—organized in a C-A-T architecture and often supplemented by additional tailoring domains [28,29,30]. For example, in the NRPSs of fluorescent *Pseudomonas* species, the A domain first activates a specific amino acid by forming an acyl-adenylate intermediate using ATP. This activated residue is subsequently transferred to the T domain, where it is covalently attached as a thioester via the phosphopantetheinyl arm. The C domain catalyzes peptide bond formation by linking the thioester-bound amino acid to the upstream peptidyl intermediate from the preceding module [29,31].

In NRPS pathways, these enzymes form modular assembly lines that activate and couple specific amino acid substrates to construct the peptide backbone of siderophores [32]. The resulting precursor is then released from the NRPS and undergoes further modification by dedicated tailoring enzymes to generate the mature siderophore structure. Representative siderophores synthesized via the NRPS pathway include bacillibactin from *Bacillus subtilis*, enterobactin from *E. coli*, and pyoverdine from *P. aeruginosa*. For pyoverdine, its biosynthetic precursor assembly occurs in the cytoplasm, while maturation proceeds in the periplasm, where NRPSs function primarily in the assembly stage [33].

Because NRPSs synthesize only the backbone, the final maturation of siderophores requires methylation, hydroxylation, heterocyclization, and glycosylation, mediated by either embedded or standalone specialized tailoring enzymes [34]. Consequently, NRPS-derived siderophores are structurally diverse, and no universally conserved gene family governs this biosynthetic route. The gene clusters encoding NRPS-derived siderophores differ considerably among species.

### 3.2. Nonribosomal Peptide Synthetase-Independent (Nis) Pathway

The NIS pathway requires a distinct family of ATP-dependent synthetases to catalyze the reactions. NIS-derived siderophores often require multiple enzymes. These enzymes adenylate carboxylic acid substrates, capture amines or alcohols, and facilitate the displacement of reaction intermediates, promoting the condensation of carboxylic acid substrates with amine or hydroxyl nucleophiles.

In the NIS pathway, specific synthetases first activate a carboxylic acid (commonly citric acid or α-ketoglutarate) by forming an ATP-dependent acyl-adenylate [35]. This activated intermediate is then attacked by an amine or hydroxyl group, yielding an amide or ester bond. These enzymes may act once or iteratively, assembling oligomeric or macrocyclic siderophores without the covalent tethering characteristic of NRPS-dependent systems. These reactions require ATP and Mg^2+^ as cofactors. The NIS pathway typically produces hydroxamate or carboxylate-type siderophores [11], including aerobactin from *E. coli* and achromobactin from *Pseudomonas* spp. [36].

Unlike the NRPS pathway, NIS gene clusters can be systematically classified into three main families (type A, B, C) and two subclasses (type A’ and C’) [29]. Type A enzymes, exemplified by IucA, are specific for citric acid; type C enzymes, exemplified by IucC, are specific for citric or succinic acid-derivatives; and type B enzymes are specific for α-ketoglutaric acid [27]. The main differences between the NRPS and NIS pathways are summarized in Table 2.

## 4. Siderophore Transport in Microorganisms

Because Fe(III)–siderophore complexes are too large to diffuse through porin channels, their transport requires specific energy-dependent carrier proteins [43]. These carriers are selective for particular Fe(III)–siderophore complexes. Siderophore transport comprises two processes: uptake and secretion [44,45]. Due to differences in cellular architecture, siderophore uptake mechanisms differ between Gram-negative and Gram-positive bacteria.

### 4.1. Siderophore Transport System in Gram-Negative Bacteria

Gram-negative bacteria possess a three-layered cell envelope structure consisting of the outer membrane, the periplasmic space, and the inner membrane. During transport, each Fe(III)–siderophore complex is initially recognized by specific outer membrane receptors and sequentially transferred by TonB-dependent transporters (TBDTs). These TBDTs are highly diverse and interact with periplasmic binding proteins (PBPs). The TonB complex consists of TonB, ExbB and ExbD proteins. TonB spans the periplasm, associates with TBDT, and links to inner membrane proteins, ExbB and ExbD [6,46,47]. In most Gram-negative bacteria, iron is released from siderophores inside the cell. However, in some bacteria, such as *P. aeruginosa*, iron is released from siderophores in the periplasm [48], indicating that iron release sites are not singular. The transport process is powered by the proton motive force (PMF) generated by TonB complex and by ATP hydrolyzed by ATP-binding cassette (ABC) transporter in the inner membrane [49].

**Figure 2:**
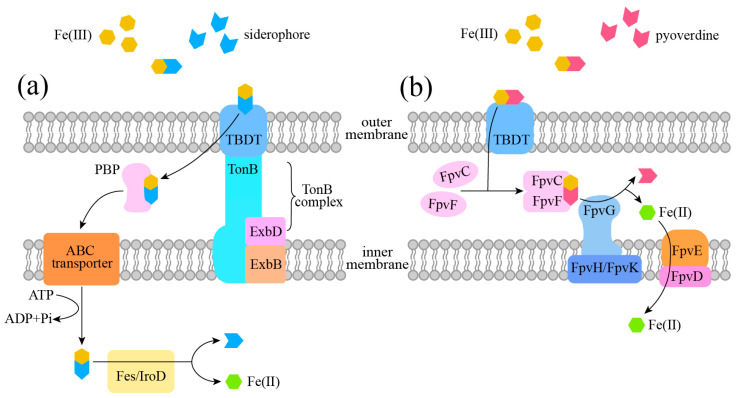
Distinct siderophore transport pathways in Gram-negative bacteria, categorized based on the site of Fe(II) release. (**a**) Cytoplasmic-release pathway. Fe(II) is released within the cytoplasm following translocation of the Fe(III)–siderophore complex across the inner membrane via TonB-dependent receptors and ABC transporters. (**b**) Periplasmic-release pathway (exemplified by pyoverdine in *P. aeruginosa*). In this mechanism, the Fe(III)–pyoverdine complex is recognized by a TBDT termed FpvA, transferred to the periplasmic FpvC-FpvF complex, and reduced in the periplasm by FpvG together with the inner membrane proteins FpvH and FpvK. The released Fe(II) is subsequently transported into the cytoplasm by the transporter FpvE-FpvD.

The transport mechanisms in Gram-negative bacteria can be summarized in five steps: (i) Under low iron stress, siderophores are secreted to chelate extracellular Fe(III). (ii) Driven by the PMF, these Fe(III)–siderophore complexes are recognized by specific TonB-dependent receptors and transported into the periplasm. (iii) PBPs shuttle the complexes across the inner membrane via ABC transporters. (iv) Fe(III) is reduced to Fe(II) by reductases located in the periplasm, inner membrane, or cytoplasm. (v) In the cytoplasm, hydrolases or esterases (e.g., Fes and IroD for catecholate siderophores) hydrolyze the complexes to release Fe(II), and siderophores are degraded or recycled (Figure 2) [50].

During transport, iron reduction can occur in the periplasm, at the inner membrane, or in the cytoplasm, where distinct reductases mediate these processes. Specifically, IroE is a periplasmic esterase; IroD is a cytoplasmic hydrolase similar to Fes [51]; FpvG is an inner-membrane reductase that requires FpvJ in the periplasm and FpvH/FpvK in the inner membrane for its full activity [52].

### 4.2. Siderophore Transport System in Gram-Positive Bacteria

By contrast, Gram-positive bacteria possess a single cytoplasmic membrane. Due to the absence of an outer membrane, Gram-positive species do not employ the TBDT-TonB-ExbBD system for siderophore uptake [53]. Instead, they utilize membrane-anchored lipoprotein siderophore-binding proteins (SBPs) to recognize and deliver Fe(III)–siderophore complexes [54]. These SBPs facilitate the internalization of ferric siderophores via a shuttle mechanism or through association with permeases. In the shuttle mechanism, Fe(III)–siderophore complexes bound to SBPs are delivered to ABC transporters [54]. In the alternative mechanism, the SBP–permease complex undergoes a conformational change to facilitate the transport across the membrane and into the cytoplasm [55]. Following internalization, Fe(III) is released from the siderophore through reduction or ligand exchange, while the siderophore molecule is degraded or recycled depending on the organism.

### 4.3. Siderophore Transport System in Fungi

Fungi acquire iron either via siderophore-mediated pathways or through reductive uptake systems [56]. The acquisition can be categorized into four types: shuttle, esterase–reductase, direct-transfer, and reductive mechanism. The shuttle and esterase–reductase mechanisms involve the internalization of siderophore–Fe(III) complexes, whereas in the direct-transfer and reductive mechanisms, iron is taken up independently of siderophores [57]. The shuttle mechanism is responsible for siderophore uptake in most common fungi.

Historically, fundamental knowledge of fungal siderophore uptake mainly derives from studies on *Saccharomyces cerevisiae* [58], which does not synthesize siderophores but appropriates those secreted by other species [59]. The uptake of xenosiderophore in *S. cerevisiae* depends on Arn/Sit transporters, which are members of the major facilitator superfamily [60]. In recent years, siderophore-mediated iron acquisition has been studied in most detail in *Aspergillus fumigatus*, where the major transporters for its siderophores have been identified [61]. The siderophore iron transporter (SIT) family in *A. fumigatus* comprises core members including Sit1, Sit2, MirB, MirC, and MirD. These members appear to be universally conserved in fungi, as their homologs have been found in many other fungi [62]. Accordingly, SITs are pivotal for fungal siderophore uptake, particularly of hydroxamate-type siderophores [63]. In biocontrol fungus *Beauveria bassiana*, two SITs, BbMirA and BbMirB, have been verified to promote fungal proliferation in vivo during the insect-colonizing stage [64]. This highlights a novel aspect of iron metabolism in fungal pathogens relevant to biocontrol.

Although the molecular characterization of specific siderophore transporters in fungi is advanced, it has not yet been integrated into a mechanistic paradigm equivalent to the stepwise model established for bacteria. This presents an opportunity to define the complete pathway from extracellular recognition to intracellular iron release, delineating its functional modules.

## 5. The Regulation of Siderophore Biosynthesis and Transport

Regulators that control siderophore biosynthesis often also modulate siderophore transport; this coordinated regulation is crucial for cellular iron homeostasis.

### 5.1. Ferric Uptake Regulator (Fur)

Fur serves as the central regulator of bacterial iron homeostasis. It forms a Fur-Fe(II) complex that binds to specific DNA sequences (Fur boxes), thereby inhibiting the transcription of siderophore biosynthesis and transport genes [65,66]. Under iron sufficiency, Fur-mediated repression protects cells from the toxicity of iron overload and reactive oxygen species (ROS) generated via the Fenton and Haber–Weiss reactions [67]. For example, Fur represses the transcription of ABC transporter genes, such as *feuABC*, to modulate siderophore uptake. In some systems, these transport modules form cell-surface signaling systems that tightly link the detection of extracellular siderophores to the induction of transport operons [68]. Conversely, under iron deficiency, a decrease in Fur-Fe(II) complexes results in the derepression of iron-regulated promoters, consequently inducing siderophore biosynthesis and uptake [69,70].

**Figure 3:**
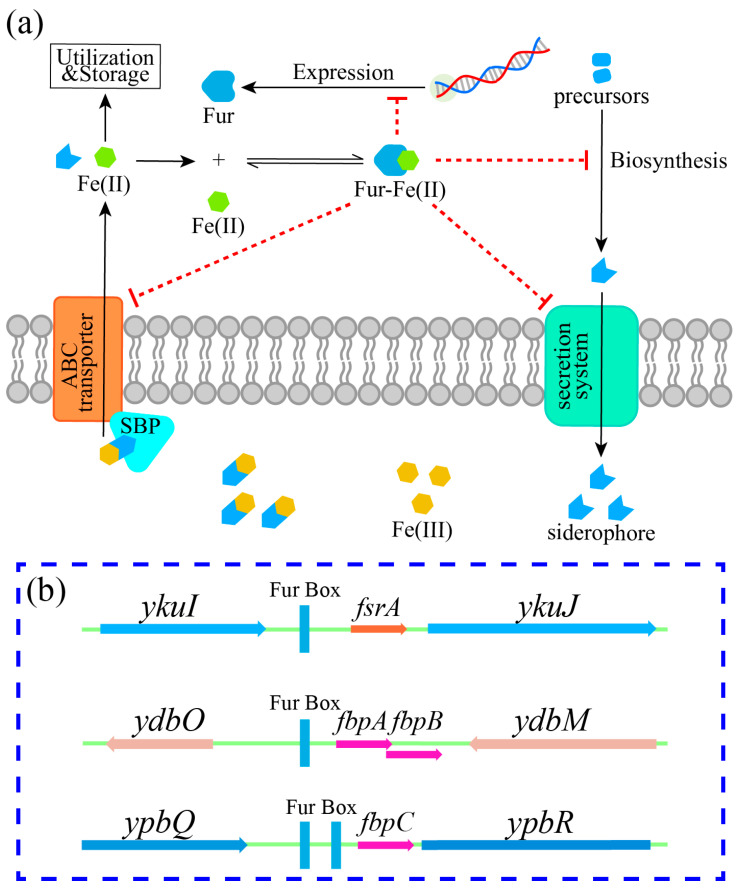
Fur-mediated regulation of siderophore biosynthesis and transport in *B. subtilis*. (**a**) Under low iron conditions, Fur becomes inactive, relieving repression of genes required for siderophore biosynthesis and transport. Under high-iron conditions, Fur binds excess Fe(II) and represses siderophore-related gene transcription while facilitating iron utilization and inducing the expression of iron storage proteins. The dashed red lines indicate indirect repression. (**b**) Genes involved in the Fur-mediated iron-sparing response in *B. subtilis*. This panel depicts the organization of the *fsrA*, *fbpAB*, and *fbpC* transcription units [71].

In *B. subtilis*, Fur mediates an iron-sparing response, ensuring that scarce iron is preferentially allocated to essential enzymes during iron starvation (Figure 3). This response is controlled by the Fur-regulated small RNA FsrA and the small proteins FbpA, FbpB, FbpC [71]. These factors repress the expression of iron-dependent proteins (e.g., succinate dehydrogenase and cytochromes) to prioritize iron for essential functions [66]. At high iron levels, this response promotes the expression of iron storage proteins but represses siderophore biosynthesis [72]. Genome-wide studies have identified additional Fur-regulated genes in *B. subtilis*, including *ypbQ*, *ykuL*, *ybpR*, and *ydbO*, which contribute to siderophore production, uptake, or broader iron homeostasis [73,74].

### 5.2. GATA-Type and Hap4-like Regulators

Instead of Fur, fungi rely on GATA-type and Hap4-like regulators to coordinate siderophore biosynthesis and iron utilization [75,76]. GATA-type repressors include SreA, Sfu1, Fep1, and Cir1. SreA, Sfu1, and Fep1 contain two GATA-type zinc fingers that are required for high-affinity DNA binding [61], whereas Cir1 harbors one zinc finger [77]. GATA-type regulators repress iron uptake and siderophore production to prevent iron toxicity under iron sufficiency but relieve this repression under iron starvation [78,79].

In *Schizosaccharomyces pombe* and some fungal pathogens, Hap4-like regulators economize iron utilization during iron starvation. Representatives of these regulators include Php4 in *S. pombe*, Hap43 in *Candida albicans*, and HapX in *Aspergillus nidulans* and *A. fumigatus* [77]. HapX, a bZIP transcriptional activator of siderophore biosynthetic genes (e.g., *sid* gene cluster), harbors four phylogenetically conserved cysteine-rich regions (CRRs). During iron starvation, it represses iron-consuming processes while activating siderophore biosynthesis and iron uptake [78,79]. Recently, a novel model has been applied to elucidate the functional dynamics of HapX in *A. fumigatus*. The four CRRs modulate their propensities to coordinate [2Fe-2S] clusters, consequently shifting HapX functions under iron starvation, iron sufficiency, and iron excess [80].

Except for *S. cerevisiae*, orthologs of SreA and HapX can be found in most fungi. *S. cerevisiae* and its related species utilize Aft1/2 and Cth1/2 as functional counterparts [63]. In *C. albicans*, *Aspergillus* sp., and *Fusarium oxysporum*, HapX forms an antagonistic regulatory circuit with SreA to maintain iron homeostasis [81,82,83]. However, interactions among other GATA-type and Hap4-like regulators beyond SreA and HapX have yet to been elucidated. Given that regulatory networks consisting of these regulators are reported in various human and plant pathogenic fungi, their in-depth study will facilitate the development of novel control strategies against these pathogens.

### 5.3. Extracytoplasmic Sigma Factor (ECF)

In the current regulatory model, ECF sigma factors are regulated by cell surface signaling (CSS) to maintain bacterial metal homeostasis [84]. For iron homeostasis, classical ECF sigma factors include PvdS, FpvI, and FecI, three iron starvation-responsive factors whose expression is repressed by Fur under iron-replete conditions.

In *Pseudomonas* sp., PvdS and FpvI regulate pyoverdine synthesis and uptake genes [85]. Binding of the Fe(III)-pyoverdine complex to its receptor FpvA transmits a signal via FpvR to trigger the derepression of PvdS and FpvI [86]. PvdS then activates pyoverdine synthesis. In parallel, FpvI controls the *fpv* gene cluster encodes membrane and periplasmic proteins that reduce siderophore-bound iron in the periplasm and mediate Fe(II) transport via the ABC transporter FpvDE [70,87].

In *E. coli*, the uptake of Fe(III)-citrate is controlled by a classical CSS system composed of the TonB-dependent receptor FecA, the ECF σ factor FecI, and the anti-sigma factor FecR [84]. Binding of Fe(III)-citrate and FecA triggers conformational changes that couple TBDT to activation of FecI via FecR, thereby inducing transcription of the *fecIR* and *fecABCDE* operons encoding the complete uptake machinery.

Notably, multiple ECF sigma factors have been reported. The putative siderophore receptor genes in *Bordetella bronchiseptica*, *bfrZ* and *bfrH*, are regulated by ECF sigma factor BupI (a homolog of *E. coli* FecI) [88] and EcfI [89]. Recently, three novel ECF sigma factors have been identified. OrbS mediates the secretion of the siderophore ornibactin in *Burkholderia cenocepacia* [90]. AsbI, an ECF sigma factor that probably lacks an dedicated anti-sigma factor, transcriptionally regulates transport of the siderophore acinetobactin in *Aeromonas salmonicida* [91]. Additionally, in *Stenotrophomonas maltophilia*, HemI has been shown to govern hemin acquisition and modulate antibiotic susceptibility [92].

### 5.4. Other Regulators

Other regulators of iron homeostasis have been reported, though they are less conserved across species. IdeR, a member of the diphtheria toxin regulator (DtxR) family, has been found in *Mycobacterium tuberculosis* and some high-GC-content Gram-positive bacteria [93]. It binds Fe(II) and represses the *mbt* operon that encodes enzymes for mycobactin biosynthesis [94]. PfeS/PfeR, belonging to the two-component systems, directly senses ferric enterobactin and activates the *pfeA* receptor gene, thereby ensuring siderophore-specific transport [95]. Additionally, local transcriptional regulators, such as the AraC-type regulator PchR, modulate expression of ferripyochelin transporters in response to siderophore binding [96].

To adapt to varying environments, microorganisms often produce multiple siderophores to ensure efficient and stable iron metabolism. A classical example is *P*. *aeruginosa*, which primarily synthesize the high-affinity pyoverdine but can switch to the low-affinity, metabolically inexpensive pyochelin under severe iron limitations [97]. This adaptive strategy is thought to minimize energy expenditure under extreme iron stress. However, the regulatory mechanisms that balance the synthesis of different siderophores await further elucidation.

### 5.5. Regulation Mediated by Quorum Sensing

In addition to transcriptional regulation, quorum sensing also contributes to siderophore production [43]. Because siderophores function as public goods, quorum sensing serves to integrate population density into the regulation of their biosynthesis and uptake. However, these siderophores can be exploited by other species for their exclusive benefit, thereby disrupting cooperative dynamics [98]. Thus, quorum sensing is a mechanism that facilitates cooperative sharing yet must contend with such ‘cheating’ behavior [99]. Consequently, the ability to the most appropriate siderophores in response to iron availability reduces energy inefficiency [100].

## 6. Preparation of Siderophore from Microorganisms

To support research and applications, gram-scale production of siderophores is required. Studies show that screening microbial strains with high siderophore yield and optimizing fermentation conditions can markedly enhance production. Based on insights into the regulatory mechanisms of natural biosynthesis, genetic engineering—such as knocking out repressor genes or modifying synthesis pathways—can be implemented to increase siderophore yield [101]. In recent years, novel siderophore engineering strategies, including precursor-directed biosynthesis, enzyme engineering, and heterologous natural product biosynthesis, have advanced siderophore preparation [2]. These methods lay a promising foundation for future scalable siderophore production.

After large-scale production, siderophores can be isolated and purified. Siderophore purification primarily relies on adsorption-based separation. Physical adsorption methods, which are generally fast and reversible, employ resin (e.g., XAD-1600 [102]) and dextran gel (e.g., Sephadex LH-20 [103]) as adsorbent materials. Chemical adsorption methods, slower but more selective, primarily involve metal chelation affinity chromatography [104]. One example of chemical adsorption is immobilized metal ion affinity chromatography (IMAC), a method that utilizes surface functional groups to specifically interact with metal ions [105]. Similarly, amino acid residues such as histidine and tryptophan on protein surfaces can interact with metal ions to form stable chelates via ligand binding—a principle that is also applied in chemical separation [106].

## 7. Application of Siderophores in Different Fields

### 7.1. Applications in Medicine

**Figure 4:**
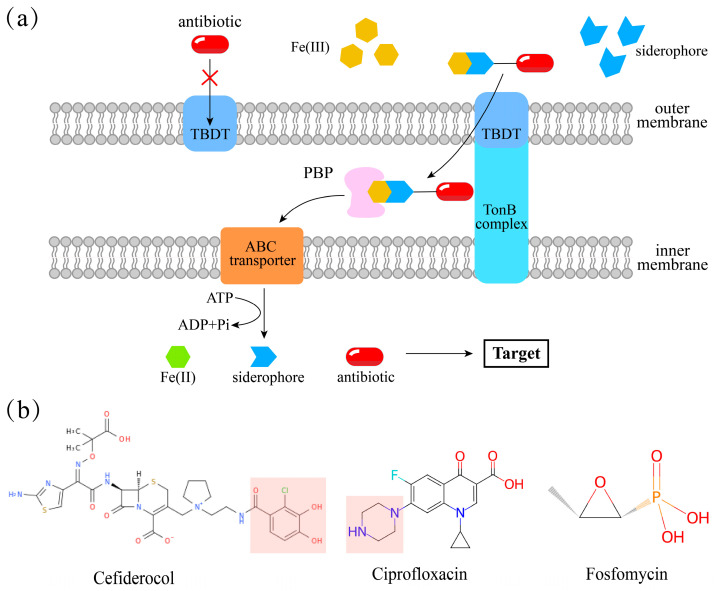
The “Trojan horse” strategy for antibiotic delivery and representative drugs. (**a**) Schematic illustration of how drugs exploit the Trojan horse strategy in Gram-negative bacteria. (**b**) Antibiotics that act by forming conjugates with siderophores via the Trojan horse strategy. Cefiderocol is a typical antibiotic of the Trojan horse strategy. Beyond their intrinsic activity, ciprofloxacin and fosfomycin can be utilized via the Trojan horse strategy through their incorporation into siderophore–antibiotic conjugates [121,122]. Structures adapted from the ChEBI database.

Siderophores can transform non-antibiotic compounds into potent antibacterial agents. For example, artemisinin conjugated to mycobactin selectively inhibits *Mycobacterium tuberculosis* [107]. This concept is termed the “Trojan horse” strategy [108]. In nature, bacteria synthesize siderophore–antibiotic conjugates (sideromycins) through this strategy to hijack the iron-uptake systems of competitors. In medicine, drugs designed on this principle are conjugated to a siderophore, mimicking natural Fe(III) to be transported into pathogens via their iron uptake systems [109,110] (Figure 4). The “Trojan horse” strategy has facilitated the development of clinical antibiotic candidates, such as GT-1 (siderophore cephalosporin) [111], BAL30072 (siderophore sulfactam) [112], and cefiderocol (siderophore cephalosporin) [113]. However, GT-1 and BAL30072 failed clinical translation due to adverse effects. Cefiderocol became the first approved siderophore–antibiotic conjugate, establishing a new antibiotic class [53] and providing a new option against antimicrobial resistance (Figure 4).

Beyond antibiotics, siderophores have applications in iron intoxication, sickle cell anemia, malaria, and cancer therapy [114,115]. Iron intoxication, often caused by excessive ingestion of ferrous salts, can be treated with siderophores that chelate iron to form water-soluble complexes such as ferrioxamine, which are then excreted in urine and feces [116]. In cancer therapy, desferrioxamine significantly suppresses the growth of aggressive tumor cells, which require higher iron levels than normal cells [117]. Siderophores show promise as vaccine components, as demonstrated in mouse experiments [118]. Nevertheless, any practical application must be substantiated by extensive clinical testing.

Siderophores also enable new diagnosis technologies based on fluorescent or radioactive signaling. Radiolabeled fungal siderophores, such as triacetylfusarinine C (TAFC) and ferricrocin (FC), are not utilized by human cells but are taken up by pathogenic fungus *A. fumigatus*. When labeled with isotopes such as ^68^Ga, they enable positron emission tomography (PET) imaging. These probes demonstrate selective uptake and strong correlation between lung signals and infection severity in preclinical models [119,120]. Thus, siderophores hold promise as highly specific diagnostic tracers.

### 7.2. Applications in Agriculture

Iron is an essential micronutrient for plant growth. Additionally, iron homeostasis genes contribute to plant immunity. Siderophores supply iron, enhancing the growth of crops, such as tomato, pea, and *Malus baccata* [123,124,125]. Plants can acquire iron through phytosiderophores or microbial siderophores. Some gramineous plants, such as rice (*Oryza sativa*), synthesize phytosiderophores for iron acquisition [126]. When phytosiderophores are insufficient, plants utilize microbial siderophores in the rhizosphere, highlighting their agricultural importance [127]. Another promising agricultural application is the use of siderophores to enhance the iron content of crops, thereby producing iron-rich foods to address nutritional deficiencies. This inexpensive biofortification strategy, distinct from crossbreeding or genetic modification, warrants further development to address malnutrition in regions with dietary iron deficiencies [128]. By enhancing plant iron nutrition and stress resilience, microbial siderophores offer a sustainable biotechnology to improve crop performance under climate change-induced stresses such as drought and soil salinity.

Fierce competition for iron resources occurs frequently among microorganisms and between microorganisms and plants. In these interactions, siderophores play a pivotal role. In the rhizosphere, siderophores from beneficial microbes can compete for iron, reducing its availability to plant pathogens [129]. Beneficial microorganisms produce siderophores that are not utilizable by pathogens, thereby exerting antagonistic effects [127]. However, this siderophore-mediated antagonism may be pronounced only under iron-limited conditions. Siderophore-mediated interactions between inoculated microbial consortia and pathogens hold promise for predicting and even suppressing phytopathogen invasions [130]. However, the feasibility of this approach requires further verification through field trials. Furthermore, iron may be essential for the biosynthesis of certain secondary metabolites with antibiotic activity, such as cyanide [131]. Thus, siderophores indirectly contribute to the antagonism against phytopathogens, as demonstrated by a study on the control of *Cephalosporium maydis* in maize [128].

### 7.3. Applications in Phytoremediation and Environmental Protection

In addition to iron, siderophores can form stable complexes with Al, Cd, Cu, Ga, In, Pb, Zn, and even radioactive metals such as U and Np [132,133,134]. By altering the oxidation state of these heavy metals, siderophores can reduce their toxicity [135]. Thus, siderophore-producing bacteria (SPB) resistant to environmental heavy metals are promising agents for phytoremediation, facilitating metal accumulation in plant tissues and enhancing plant metal tolerance. Hydroxamate siderophores produced by SPB can also protect microbial auxins that can be utilizable by plants from oxidative degradation [8]. Moreover, siderophores have been proposed for separating U and Pu from nuclear waste for reuse, offering potential benefits for the nuclear industry [136].

Beyond terrestrial applications, siderophores produced by marine microorganisms (e.g., petrobactin produced by *Marinobacter hydrocarbonoclasticus*) exhibit potential in mitigating marine pollution by indirectly promoting the biodegradation of petroleum hydrocarbons [137]. However, further research is needed to translate these findings into practical applications.

### 7.4. Applications in Biosensing

The metal-binding specificity of siderophores makes them attractive for biosensor applications. Natural siderophores such as 2,3-dihydroxybenzoylglycine can be anchored onto Fe_3_O_4_ nanoparticles to form nano-biosensors (HL-FeNPs), which enables highly selective fluorescence-based detection of Al^3+^ at nanomolar concentrations in aqueous solution [138]. Fluorescent siderophores, including pyoverdine, have been used to fabricate nanosensors that detect metal ions, such as Cu^2+^, for environmental monitoring of copper pollution in aquatic environments [139,140]. Siderophore-based biosensors can detect pesticide residues through fluorescence quenching. Pyoverdine from *P. aeruginosa* strain PA1 can be used to detect furazolidone, a pesticide with high environmental persistence and strong genotoxicity [141].

For whole-cell microbial detection, siderophores have served as recognition ligands in localized surface plasmon resonance (LSPR) biosensors to selectively capture microorganisms such as *Acinetobacter baumannii* [142]. In medical settings, immobilized siderophores on platforms such as gold chips enable rapid and species-specific pathogen detection, although limitations related to cross-reactivity remain to be addressed [143,144].

## 8. Conclusions

Siderophores represent an essential adaptive strategy that enables microorganisms to acquire iron under limiting conditions and in fluctuating redox environments. Microorganisms achieve this through a highly regulated biosynthesis (via NRPS and NIS pathways) and uptake, which is governed by diverse iron-responsive regulators to maintain iron homeostasis. Although the molecular machinery for siderophore transport differs between Gram-negative and Gram-positive bacteria, it is universally a complex, energy-dependent process reliant on dedicated receptor systems to sense and internalize ferric complexes.

Ecologically, siderophores shape microbial community dynamics by mediating both cooperation and competition for iron. They enable cooperative iron sharing but also drive competitive interactions through siderophore piracy and receptor-mediated exclusion. These dynamics highlight siderophores as central mediators of microbial interactions and adaptation to changing environmental conditions, including those driven by climate change.

Beyond their ecological roles, siderophores exhibit broad potential in sustainable biotechnology. In medicine, they serve as valuable agents for antimicrobial and iron-chelation therapies. Thus, siderophores represent a promising approach to overcoming resistance within a One Health framework linking human and animal health. Their high-affinity metal-binding properties support bioremediation of contaminated soils and waters, while in sustainable agriculture, they contribute to climate-resilient, low-input systems by improving iron nutrition and biological control. Collectively, these properties position siderophores at the intersection of microbiology, sustainable biotechnology, environmental resilience, and the One Health concept.

Despite substantial progress, fully exploiting siderophores requires overcoming key technical and ecological constraints. Optimization of fermentation and downstream processing remains essential for economically and environmentally sustainable large-scale production. Exploration of extreme environments and host-associated microbiomes, many of which are sensitive to climate change, may yield novel siderophores with distinctive biochemical traits. Integrative approaches combining genomics, metabolic engineering, and synthetic biology are key to comprehensively elucidating siderophore regulation and transport across environmental, agricultural, and clinical contexts. Ultimately, such interdisciplinary efforts will connect mechanistic insights with applications in medicine, agriculture, and environmental management, supporting sustainable development under the One Health and climate change frameworks.

## Figures and Tables

**Table 2:** Main differences between NRPS and NIS pathways.

Features	NRPS Pathway	NIS Pathway	References
Main Enzymes	Nonribosomal peptide synthetases (NRPSs)	NRPS-independent synthetases	[27]
Precursors	Primarily amino acids	Small carboxylic acids (citrate, succinate), polyamines or hydroxylamines (hydroxylated lysine, putrescine)	[27,30]
Assembly Logic	Thiotemplate modular assembly with colinear addition of building blocks	ATP-dependent condensation of carboxylate and nucleophile independent of thiotemplate-based carrier protein domains	[35]
Reported Taxonomic Occurrence	Widely reported in bacteria and fungi	Characterized in bacteria; fewer reports in fungi.	[29]
Typical Products and Relevant Gene Clusters	Bacillibactin: *dhbA-F* Enterobactin: *entA-F* Pyoverdine: *pvdDIJKL* Mycobactin: *mbtA-J*	Aerobactin: *iucA-D* Desferrioxamine: *desA-D* Achromobactin: *acsACD* Petrobactin: *asbAB*	[27,28,29,37,38,39,40,41,42]

## Data Availability

No new data were created or analyzed in this study. Data sharing is not applicable to this article.

## References

[1] Kloepper J.W., Leong J., Teintze M., Schroth M.N. (1980). Enhanced plant growth by siderophores produced by plant growth-promoting rhizobacteria. Nature.

[2] Puja H., Mislin G.L.A., Rigouin C. (2023). Engineering Siderophore Biosynthesis and Regulation Pathways to Increase Diversity and Availability. Biomolecules.

[3] Neilands J.B. (1995). Siderophores: Structure and Function of Microbial Iron Transport Compounds. J. Biol. Chem..

[4] He R., Gu S., Xu J., Li X., Chen H., Shao Z., Wang F., Shao J., Yin W.B., Qian L. (2024). SIDERITE: Unveiling hidden siderophore diversity in the chemical space through digital exploration. iMeta.

[5] Soares E.V. (2022). Perspective on the biotechnological production of bacterial siderophores and their use. Appl. Microbiol. Biotechnol..

[6] Timofeeva A.M., Galyamova M.R., Sedykh S.E. (2022). Bacterial siderophores: Classification, biosynthesis, perspectives of use in agriculture. Plants.

[7] Upritchard H.G., Yang J., Bremer P.J., Lamont I.L., McQuillan A.J. (2011). Adsorption of Enterobactin to Metal Oxides and the Role of Siderophores in Bacterial Adhesion to Metals. Langmuir.

[8] Rajkumar M., Ae N., Prasad M.N.V., Freitas H. (2010). Potential of siderophore-producing bacteria for improving heavy metal phytoextraction. Trends Biotechnol..

[9] Winkelmann G. (2007). Ecology of siderophores with special reference to the fungi. BioMetals.

[10] Miethke M., Marahiel M.A. (2007). Siderophore-Based Iron Acquisition and Pathogen Control. Microbiol. Mol. Biol. Rev..

[11] Barry S.M., Challis G.L. (2009). Recent advances in siderophore biosynthesis. Curr. Opin. Chem. Biol..

[12] Günter K., Toupet C., Schupp T. (1993). Characterization of an iron-regulated promoter involved in desferrioxamine B synthesis in *Streptomyces pilosus*: Repressor-binding site and homology to the diphtheria toxin gene promoter. J. Bacteriol..

[13] Penwell W.F., DeGrace N., Tentarelli S., Gauthier L., Gilbert C.M., Arivett B.A., Miller A.A., Durand-Reville T.F., Joubran C., Actis L.A. (2015). Discovery and Characterization of New Hydroxamate Siderophores, Baumannoferrin A and B, produced by *Acinetobacter baumannii*. ChemBioChem.

[14] Aguiar M., Orasch T., Shadkchan Y., Caballero P., Pfister J., Sastré-Velásquez L.E., Gsaller F., Decristoforo C., Osherov N., Haas H. (2022). Uptake of the Siderophore Triacetylfusarinine C, but Not Fusarinine C, Is Crucial for Virulence of *Aspergillus fumigatus*. mBio.

[15] Page W.J., Tigerstrom M.V. (1988). Aminochelin, a Catecholamine Siderophore Produced by *Azotobacter vinelandii*. Microbiology.

[16] Ciche T.A., Blackburn M., Carney J.R., Ensign J.C. (2003). Photobactin: A Catechol Siderophore Produced by *Photorhabdus luminescens*, an Entomopathogen Mutually Associated with *Heterorhabditis bacteriophora* NC1 Nematodes. Appl. Environ. Microbiol..

[17] Zheng T., Nolan E.M. (2014). Enterobactin-mediated delivery of β-lactam antibiotics enhances antibacterial activity against pathogenic *Escherichia coli*. J. Am. Chem. Soc..

[18] Lam M.M.C., Wyres K.L., Judd L.M., Wick R.R., Jenney A., Brisse S., Holt K.E. (2018). Tracking key virulence loci encoding aerobactin and salmochelin siderophore synthesis in *Klebsiella pneumoniae*. Genome Med..

[19] Rabbee M.F., Ali M.S., Choi J., Hwang B.S., Jeong S.C., Baek K.-h. (2019). *Bacillus velezensis*: A Valuable Member of Bioactive Molecules within Plant Microbiomes. Molecules.

[20] Smith M.J., Shoolery J.N., Schwyn B., Holden I., Neilands J.B. (1985). Rhizobactin, a structurally novel siderophore from *Rhizobium meliloti*. J. Am. Chem. Soc..

[21] Beasley F.C., Vinés E.D., Grigg J.C., Zheng Q., Liu S., Lajoie G.A., Murphy M.E.P., Heinrichs D.E. (2009). Characterization of staphyloferrin A biosynthetic and transport mutants in *Staphylococcus aureus*. Mol. Microbiol..

[22] Mo K.-F., Dai Z., Wunschel D.S. (2016). Production and Characterization of Desmalonichrome Relative Binding Affinity for Uranyl Ions in Relation to Other Siderophores. J. Nat. Prod..

[23] Ørskov I., Svanborg Eden C., Ørskov F. (1987). Aerobactin production of serotyped *Escherichia coli* from urinary tract infections. Med. Microbiol. Immunol..

[24] Meena B., Radhajeyalakshmi R., Marimuthu T., Vidhyasekaran P., Doraiswamy S., Velazhahan R. (2000). Induction of pathogenesis-related proteins, phenolics and phenylalanine ammonia-lyase in groundnut by *Pseudomonas fluorescens*. J. Plant Dis. Prot..

[25] Carrano C.J., Jordan M., Drechsel H., Schmid D.G., Winkelmann G. (2001). Heterobactins: A new class of siderophores from *Rhodococcus erythropolis* IGTS8 containing both hydroxamate and catecholate donor groups. Biometals.

[26] Stintzi A., Raymond K.N., Templeton D., Templeton D. (2002). Siderophore chemistry. Molecular Cellular Iron Transport.

[27] Oves-Costales D., Kadi N., Challis G.L. (2009). The long-overlooked enzymology of a nonribosomal peptide synthetase-independent pathway for virulence-conferring siderophore biosynthesis. Chem. Commun..

[28] Mossialos D., Ochsner U., Baysse C., Chablain P., Pirnay J.P., Koedam N., Budzikiewicz H., Fernández D.U., Schäfer M., Ravel J. (2002). Identification of new, conserved, non-ribosomal peptide synthetases from fluorescent pseudomonads involved in the biosynthesis of the siderophore pyoverdine. Mol. Microbiol..

[29] Carroll C.S., Moore M.M. (2018). Ironing out siderophore biosynthesis: A review of non-ribosomal peptide synthetase (NRPS)-independent siderophore synthetases. Crit. Rev. Biochem. Mol. Biol..

[30] Felnagle E.A., Jackson E.E., Chan Y.A., Podevels A.M., Berti A.D., McMahon M.D., Thomas M.G. (2008). Nonribosomal Peptide Synthetases Involved in the Production of Medically Relevant Natural Products. Mol. Pharm..

[31] Konz D., Marahiel M.A. (1999). How do peptide synthetases generate structural diversity?. Chem. Biol..

[32] He R., Zhang J., Shao Y., Gu S., Song C., Qian L., Yin W.-B., Li Z. (2023). Knowledge-guided data mining on the standardized architecture of NRPS: Subtypes, novel motifs, and sequence entanglements. PLoS Comput. Biol..

[33] Ringel M.T., Brüser T. (2018). The biosynthesis of pyoverdines. Microb. Cell.

[34] Walsh C.T., Chen H., Keating T.A., Hubbard B.K., Losey H.C., Luo L., Marshall C.G., Miller D.A., Patel H.M. (2001). Tailoring enzymes that modify nonribosomal peptides during and after chain elongation on NRPS assembly lines. Curr. Opin. Chem. Biol..

[35] Patel K.D., Fisk M.B., Gulick A.M. (2024). Discovery, functional characterization, and structural studies of the NRPS-independent siderophore synthetases. Crit. Rev. Biochem. Mol. Biol..

[36] Blanco Nouche C., Paris C., Dhalleine T., Oger P., Turpault M.-P., Uroz S. (2023). The non-ribosomal peptide synthetase-independent siderophore (NIS) rhizobactin produced by *Caballeronia mineralivorans* PML1(12) confers the ability to weather minerals. Appl. Environ. Microbiol..

[37] Quadri L.E.N., Sello J., Keating T.A., Weinreb P.H., Walsh C.T. (1998). Identification of a *Mycobacterium tuberculosis* gene cluster encoding the biosynthetic enzymes for assembly of the virulence-conferring siderophore mycobactin. Chem. Biol..

[38] May J.J., Wendrich T.M., Marahiel M.A. (2001). The *dhb* Operon of *Bacillus subtilis* Encodes the Biosynthetic Template for the Catecholic Siderophore 2,3-Dihydroxybenzoate-Glycine-Threonine Trimeric Ester Bacillibactin. J. Biol. Chem..

[39] Tunca S., Barreiro C., Sola-Landa A., Coque J.J.R., Martín J.F. (2007). Transcriptional regulation of the desferrioxamine gene cluster of *Streptomyces coelicolor* is mediated by binding of DmdR1 to an iron box in the promoter of the *desA* gene. FEBS J..

[40] Manck L.E., Park J., Tully B.J., Poire A.M., Bundy R.M., Dupont C.L., Barbeau K.A. (2022). Petrobactin, a siderophore produced by Alteromonas, mediates community iron acquisition in the global ocean. ISME J..

[41] Mydy L.S., Bailey D.C., Patel K.D., Rice M.R., Gulick A.M. (2020). The Siderophore Synthetase IucA of the Aerobactin Biosynthetic Pathway Uses an Ordered Mechanism. Biochemistry.

[42] Berti A.D., Thomas M.G. (2009). Analysis of Achromobactin Biosynthesis by Pseudomonas syringae pv. syringae B728a. J. Bacteriol..

[43] Hider R.C., Kong X. (2010). Chemistry and biology of siderophores. Nat. Prod. Rep..

[44] Raymond K.N., Allred B.E., Sia A.K. (2015). Coordination Chemistry of Microbial Iron Transport. Acc. Chem. Res..

[45] Heine T., Mehnert M., Schwabe R., Tischler D. (2017). Siderophore Purification via Immobilized Metal Affinity Chromatography. Solid State Phenom..

[46] Braun V. (2024). Substrate Uptake by TonB-Dependent Outer Membrane Transporters. Mol Microbiol.

[47] Braun V., Ratliff A.C., Celia H., Buchanan S.K. (2023). Energization of Outer Membrane Transport by the ExbB ExbD Molecular Motor. J. Bacteriol..

[48] Brillet K., Ruffenach F., Adams H., Journet L., Gasser V., Hoegy F., Guillon L., Hannauer M., Page A., Schalk I.J. (2012). An ABC Transporter with Two Periplasmic Binding Proteins Involved in Iron Acquisition in *Pseudomonas aeruginosa*. ACS Chem. Biol..

[49] Klebba P.E. (2016). ROSET model of TonB action in gram-negative bacterial iron acquisition. J. Bacteriol..

[50] Josts I., Veith K., Normant V., Schalk I.J., Tidow H. (2021). Structural insights into a novel family of integral membrane siderophore reductases. Proc. Natl. Acad. Sci. USA.

[51] Zhu M., Valdebenito M., Winkelmann G., Hantke K. (2005). Functions of the siderophore esterases IroD and IroE in iron-salmochelin utilization. Microbiology.

[52] Hantke K., Nicholson G., Rabsch W., Winkelmann G. (2003). Salmochelins, siderophores of *Salmonella enterica* and uropathogenic *Escherichia coli* strains, are recognized by the outer membrane receptor IroN. Proc. Natl. Acad. Sci. USA.

[53] Gräff Á.T., Barry S.M. (2024). Siderophores as tools and treatments. npj Antimicrob. Resist..

[54] Fukushima T., Allred B.E., Raymond K.N. (2014). Direct Evidence of Iron Uptake by the Gram-Positive Siderophore-Shuttle Mechanism without Iron Reduction. ACS Chem. Biol..

[55] Rocha B.M., Pinto E., Sousa E., Resende D.I.S.P. (2025). Targeting Siderophore Biosynthesis to Thwart Microbial Growth. Int. J. Mol. Sci..

[56] Philpott C.C. (2006). Iron uptake in fungi: A system for every source. Biochim. Biophys. Acta (BBA) Mol. Cell Res..

[57] Howard D.H. (1999). Acquisition, Transport, and Storage of Iron by Pathogenic Fungi. Clin. Microbiol. Rev..

[58] Renshaw J.C., Robson G.D., Trinci A.P.J., Wiebe M.G., Livens F.R., Collison D., Taylor R.J. (2002). Fungal siderophores: Structures, functions and applications. Mycol. Res..

[59] Mehra R.K., Winge D.R. (1991). Metal ion resistance in fungi: Molecular mechanisms and their regulated expression. J. Cell. Biochem..

[60] Stanford F.A., Voigt K. (2020). Iron Assimilation during Emerging Infections Caused by Opportunistic Fungi with emphasis on Mucorales and the Development of Antifungal Resistance. Genes.

[61] Happacher I., Aguiar M., Yap A., Decristoforo C., Haas H.A.-O. (2023). Fungal siderophore metabolism with a focus on Aspergillus fumigatus: Impact on biotic interactions and potential translational applications. Essays Biochem.

[62] Choi S., Kronstad J.W., Jung W.H. (2024). Siderophore Biosynthesis and Transport Systems in Model and Pathogenic Fungi. J. Microbiol. Biotechnol..

[63] Haas H. (2012). Iron—A Key Nexus in the Virulence of Aspergillus fumigatus. Front. Microbiol..

[64] Zhu C., Liu Q., Chen Y., Tian F., Kong D., Happacher I., Haas H., Zhang Y., Luo Z. (2025). The siderophore-iron transporter BbMirB is required for the fungal pathogen Beauveria bassiana to repress insect immunity and promote proliferation during colonization of hemocoel. mBio.

[65] Escolar L., Pérez-Martín J., De Lorenzo V. (1999). Opening the Iron Box: Transcriptional Metalloregulation by the Fur Protein. J. Bacteriol..

[66] Hantke K. (2001). Iron and metal regulation in bacteria. Curr. Opin. Microbiol..

[67] Santos R.E.R.D.S., Batista B.B., Da Silva Neto J.F. (2020). Ferric Uptake Regulator Fur Coordinates Siderophore Production and Defense against Iron Toxicity and Oxidative Stress and Contributes to Virulence in Chromobacterium violaceum. Appl. Environ. Microbiol..

[68] Llamas M.A., Imperi F., Visca P., Lamont I.L. (2014). Cell-surface signaling in *Pseudomonas*: Stress responses, iron transport, and pathogenicity. FEMS Microbiol. Rev..

[69] Venturi V., Weisbeek P., Koster M. (1995). Gene regulation of siderophore-mediated iron acquisition in *Pseudomonas*: Not only the Fur repressor. Mol. Microbiol..

[70] Cornelis P., Tahrioui A., Lesouhaitier O., Bouffartigues E., Feuilloley M., Baysse C., Chevalier S. (2023). High affinity iron uptake by pyoverdine in *Pseudomonas aeruginosa* involves multiple regulators besides Fur, PvdS, and FpvI. BioMetals.

[71] Gaballa A., Antelmann H., Aguilar C., Khakh S.K., Song K.-B., Smaldone G.T., Helmann J.D. (2008). The *Bacillus subtilis* iron-sparing response is mediated by a Fur-regulated small RNA and three small, basic proteins. Proc. Natl. Acad. Sci. USA.

[72] Ollinger J., Song K.-B., Antelmann H., Hecker M., Helmann J.D. (2006). Role of the Fur Regulon in Iron Transport in *Bacillus subtilis*. J. Bacteriol..

[73] Pi H., Helmann J.D. (2018). Genome-Wide Characterization of the Fur Regulatory Network Reveals a Link between Catechol Degradation and Bacillibactin Metabolism in *Bacillus subtilis*. mBio.

[74] Sattler L., Graumann P.L. (2021). Real-Time Messenger RNA Dynamics in *Bacillus subtilis*. Front. Microbiol..

[75] Gsaller F., Hortschansky P., Beattie S.R., Klammer V., Tuppatsch K., Lechner B.E., Rietzschel N., Werner E.R., Vogan A.A., Chung D. (2014). The Janus transcription factor HapX controls fungal adaptation to both iron starvation and iron excess. EMBO J..

[76] Haas H. (2014). Fungal siderophore metabolism with a focus on *Aspergillus fumigatus*. Nat. Prod. Rep..

[77] Gupta M., Outten C.E. (2020). Iron-sulfur cluster signaling: The common thread in fungal iron regulation. Curr. Opin. Chem. Biol..

[78] Schrettl M., Kim H.S., Eisendle M., Kragl C., Nierman W.C., Heinekamp T., Werner E.R., Jacobsen I., Illmer P., Yi H. (2008). SreA-mediated iron regulation in *Aspergillus fumigatus*. Mol. Microbiol..

[79] Schrettl M., Beckmann N., Varga J., Heinekamp T., Jacobsen I.D., Jöchl C., Moussa T.A., Wang S., Gsaller F., Blatzer M. (2010). HapX-Mediated Adaption to Iron Starvation Is Crucial for Virulence of *Aspergillus fumigatus*. PLoS Pathog..

[80] Oberegger S., Misslinger M., Happacher I., Haas H. (2025). Functional transitions of the Aspergillus fumigatus iron regulator HapX are governed by conserved domains cooperatively binding [2Fe-2S] clusters. Nucleic Acids Res.

[81] Chen C., Pande K., French S.D., Tuch B.B., Noble S.M. (2011). An Iron Homeostasis Regulatory Circuit with Reciprocal Roles in *Candida albicans* Commensalism and Pathogenesis. Cell Host Microbe.

[82] Philpott C.C., Leidgens S., Frey A.G. (2012). Metabolic remodeling in iron-deficient fungi. Biochim. Biophys. Acta-Mol. Cell Res..

[83] Lopez-Berges M.S., Capilla J., Turra D., Schafferer L., Matthijs S., Jochl C., Cornelis P., Guarro J., Haas H., Di Pietro A. (2012). HapX-mediated iron homeostasis is essential for rhizosphere competence and virulence of the soilborne pathogen *Fusarium oxysporum*. Plant Cell.

[84] Moraleda-Munoz A., Javier Marcos-Torres F., Perez J., Munoz-Dorado J. (2019). Metal-responsive RNA polymerase extracytoplasmic function (ECF) sigma factors. Mol. Microbiol..

[85] Lamont I.L., Martin L.W. (2003). Identification and characterization of novel pyoverdine synthesis genes in *Pseudomonas aeruginosa*. Microbiology.

[86] Beare P.A., For R.J., Martin L.W., Lamont I.L. (2003). Siderophore-mediated cell signalling in Pseudomonas aeruginosa: Divergent pathways regulate virulence factor production and siderophore receptor synthesis. Mol. Microbiol..

[87] Schulz S., Eckweiler D., Bielecka A., Nicolai T., Franke R., Dötsch A., Hornischer K., Bruchmann S., Düvel J., Häussler S. (2015). Elucidation of Sigma Factor-Associated Networks in *Pseudomonas aeruginosa* Reveals a Modular Architecture with Limited and Function-Specific Crosstalk. PLoS Pathog..

[88] Pradel E., Locht C. (2001). Expression of the putative siderophore receptor gene *bfrZ* is controlled by the extracytoplasmic-function sigma factor BupI in *Bordetella bronchiseptica*. J. Bacteriol..

[89] Burgos J.M., King-Lyons N.D., Connell T.D. (2010). Expression of BfrH, a Putative Siderophore Receptor of *Bordetella bronchiseptica*, Is Regulated by Iron, Fur1, and the Extracellular Function Sigma Factor EcfI. Infect. Immun..

[90] Butt A.T., Banyard C.D., Haldipurkar S.S., Agnoli K., Mohsin M.I., Vitovski S., Paleja A., Tang Y., Lomax R., Ye F. (2022). The Burkholderia cenocepacia iron starvation σ factor, OrbS, possesses an on-board iron sensor. Nucleic Acids Res..

[91] Rey-Varela D., Balado M., Lemos M.L. (2023). The Sigma Factor AsbI Is Required for the Expression of Acinetobactin Siderophore Transport Genes in Aeromonas salmonicida. Int. J. Mol. Sci..

[92] Liao C.H., Ku R.H., Lu H.F., Hu E.W., Li L.H., Yang T.C. (2025). Involvement of HemI, an ECF sigma factor, in hemin acquisition and antibiotic susceptibility in Stenotrophomonas maltophilia. Front. Cell. Infect. Microbiol..

[93] Cheng Y., Yang R., Lyu M., Wang S., Liu X., Wen Y., Song Y., Li J., Chen Z. (2018). IdeR, a DtxR Family Iron Response Regulator, Controls Iron Homeostasis, Morphological Differentiation, Secondary Metabolism, and the Oxidative Stress Response in *Streptomyces avermitilis*. Appl. Environ. Microbiol..

[94] Sritharan M. (2016). Iron Homeostasis in *Mycobacterium tuberculosis*: Mechanistic Insights into Siderophore-Mediated Iron Uptake. J. Bacteriol..

[95] Dean C.R., Neshat S., Poole K. (1996). PfeR, an enterobactin-responsive activator of ferric enterobactin receptor gene expression in *Pseudomonas aeruginosa*. J. Bacteriol..

[96] Heinrichs D.E., Poole K. (1996). PchR, a regulator of ferripyochelin receptor gene (*fptA*) expression in *Pseudomonas aeruginosa*, functions both as an activator and as a repressor. J. Bacteriol..

[97] Dumas Z., Ross-Gillespie A., Kümmerli R. (2013). Switching between apparently redundant iron-uptake mechanisms benefits bacteria in changeable environments. Proc. R. Soc. B Biol. Sci..

[98] Niehus R., Picot A., Oliveira N.M., Mitri S., Foster K.R. (2017). The evolution of siderophore production as a competitive trait: The Competitive Evolution of Siderophores. Evolution.

[99] Özkaya Ö., Balbontín R., Gordo I., Xavier K.B. (2018). Cheating on Cheaters Stabilizes Cooperation in *Pseudomonas aeruginosa*. Curr. Biol..

[100] Poole K. (2003). Iron acquisition and its control in *Pseudomonas aeruginosa* many roads lead to rome. Front. Biosci..

[101] Liu L., Li S., Wang S., Dong Z., Gao H. (2018). Complex Iron Uptake by the Putrebactin-Mediated and Feo Systems in Shewanella oneidensis. Appl Env. Microbiol.

[102] Xu L., Han T., Ge M., Zhu L., Qian X. (2016). Discovery of the New Plant Growth-Regulating Compound LYXLF2 Based on Manipulating the Halogenase in *Amycolatopsis orientalis*. Curr. Microbiol..

[103] Patel A.K., Deshattiwar M.K., Chaudhari B.L., Chincholkar S.B. (2009). Production, purification and chemical characterization of the catecholate siderophore from potent probiotic strains of *Bacillus* spp.. Bioresour. Technol..

[104] Sayyed R., Chincholkar S. (2006). Purification of siderophores of *Alcaligenes faecalis* on Amberlite XAD. Bioresour. Technol..

[105] Ye J., Zhang X., Young C., Zhao X., Hao Q., Cheng L., Jensen O.N. (2010). Optimized IMAC−IMAC Protocol for Phosphopeptide Recovery from Complex Biological Samples. J. Proteome Res..

[106] Porath J. (1988). IMAC—Immobilized metal ion affinity based chromatography. TrAC Trends Anal. Chem..

[107] Pham T.N., Loupias P., Dassonville-Klimpt A., Sonnet P. (2019). Drug delivery systems designed to overcome antimicrobial resistance. Med. Res. Rev..

[108] Miller M.J., Walz A.J., Zhu H., Wu C., Moraski G., Möllmann U., Tristani E.M., Crumbliss A.L., Ferdig M.T., Checkley L. (2011). Design, Synthesis, and Study of a Mycobactin−Artemisinin Conjugate That Has Selective and Potent Activity against Tuberculosis and Malaria. J. Am. Chem. Soc..

[109] Ito A., Sato T., Ota M., Takemura M., Nishikawa T., Toba S., Kohira N., Miyagawa S., Ishibashi N., Matsumoto S. (2018). *In Vitro* Antibacterial Properties of Cefiderocol, a Novel Siderophore Cephalosporin, against Gram-Negative Bacteria. Antimicrob. Agents Chemother..

[110] Schalk I.J. (2018). Siderophore–antibiotic conjugates: Exploiting iron uptake to deliver drugs into bacteria. Clin. Microbiol. Infect..

[111] Halasohoris S.A., Scarff J.M., Pysz L.M., Lembirik S., Lemmon M.M., Biek D., Hannah B., Zumbrun S.D., Panchal R.G. (2021). In vitro and in vivo activity of GT-1, a novel siderophore cephalosporin, and GT-055, a broad-spectrum β-lactamase inhibitor, against biothreat and ESKAPE pathogens. J. Antibiot..

[112] Page M.G.P., Dantier C., Desarbre E. (2010). *In Vitro* Properties of BAL30072, a Novel Siderophore Sulfactam with Activity against Multiresistant Gram-Negative Bacilli. Antimicrob. Agents Chemother..

[113] Bonomo R.A. (2019). Cefiderocol: A novel siderophore cephalosporin defeating carbapenem-resistant pathogens. Clin. Infect. Dis..

[114] Górska A., Sloderbach A., Marszałł M.P. (2014). Siderophore–drug complexes: Potential medicinal applications of the ‘Trojan horse’ strategy. Trends Pharmacol. Sci..

[115] Fan D., Fang Q. (2021). Siderophores for medical applications: Imaging, sensors, and therapeutics. Int. J. Pharm..

[116] Nagoba B., Vedpathak D. (2011). Medical Applications of Siderophores. Electron. J. Gen. Med..

[117] Nakouti I., Sihanonth P., Palaga T., Hobbs G. (2013). Effect of a siderophore producer on animal cell apoptosis: A possible role as anti-cancer agent. Int. J. Pharma Med. Biol. Sci..

[118] Pecoraro L., Wang X., Shah D., Song X., Kumar V., Shakoor A., Tripathi K., Ramteke P.W., Rani R. (2021). Biosynthesis Pathways, Transport Mechanisms and Biotechnological Applications of Fungal Siderophores. J. Fungi.

[119] Thornton C.R. (2018). Molecular Imaging of Invasive Pulmonary Aspergillosis Using ImmunoPET/MRI: The Future Looks Bright. Front. Microbiol..

[120] Pfister J., Summer D., Petrik M., Khoylou M., Lichius A., Kaeopookum P., Kochinke L., Orasch T., Haas H., Decristoforo C. (2020). Hybrid Imaging of *Aspergillus fumigatus* Pulmonary Infection with Fluorescent, 68Ga-Labelled Siderophores. Biomolecules.

[121] Sanderson T.J., Black C.M., Southwell J.W., Wilde E.J., Pandey A., Herman R., Thomas G.H., Boros E., Duhme-Klair A.-K., Routledge A. (2020). A Salmochelin S4-Inspired Ciprofloxacin Trojan Horse Conjugate. ACS Infect. Dis..

[122] Khazaal M.T., Faraag A.H.I., El-Hendawy H.H. (2024). In vitro and in silico studies of enterobactin-inspired Ciprofloxacin and Fosfomycin first generation conjugates on the antibiotic resistant E. coli OQ866153. BMC Microbiol..

[123] Nagata T., Oobo T., Aozasa O. (2013). Efficacy of a bacterial siderophore, pyoverdine, to supply iron to *Solanum lycopersicum* plants. J. Biosci. Bioeng..

[124] Lurthy T., Cantat C., Jeudy C., Declerck P., Gallardo K., Barraud C., Leroy F., Ourry A., Lemanceau P., Salon C. (2020). Impact of Bacterial Siderophores on Iron Status and Ionome in Pea. Front. Plant Sci..

[125] Gao B., Chai X., Huang Y., Wang X., Han Z., Xu X., Wu T., Zhang X., Wang Y. (2022). Siderophore production in pseudomonas SP. strain SP3 enhances iron acquisition in apple rootstock. J. Appl. Microbiol..

[126] Li M., Watanabe S., Gao F., Dubos C. (2023). Iron Nutrition in Plants: Towards a New Paradigm?. Plants.

[127] Gu S., Wei Z., Shao Z., Friman V.-P., Cao K., Yang T., Kramer J., Wang X., Li M., Mei X. (2020). Competition for iron drives phytopathogen control by natural rhizosphere microbiomes. Nat. Microbiol..

[128] Ghazy N., El-Nahrawy S. (2021). Siderophore production by *Bacillus subtilis* MF497446 and *Pseudomonas koreensis* MG209738 and their efficacy in controlling *Cephalosporium maydis* in maize plant. Arch. Microbiol..

[129] Beneduzi A., Ambrosini A., Passaglia L.M.P. (2012). Plant growth-promoting rhizobacteria (PGPR): Their potential as antagonists and biocontrol agents. Genet. Mol. Biol..

[130] Gu S., Yang T., Shao Z., Wang T., Cao K., Jousset A., Friman V.-P., Mallon C., Mei X., Wei Z. (2020). Siderophore-Mediated Interactions Determine the Disease Suppressiveness of Microbial Consortia. mSystems.

[131] Voisard C., Keel C., Haas D., Dèfago G. (1989). Cyanide production by *Pseudomonas fluorescens* helps suppress black root rot of tobacco under gnotobiotic conditions. EMBO J..

[132] Neubauer U., Furrer G., Kayser A., Schulin R. (2000). Siderophores, NTA, and Citrate: Potential Soil Amendments to Enhance Heavy Metal Mobility in Phytoremediation. Int. J. Phytoremediation.

[133] Kiss T., Farkas E. (1998). Metal-binding Ability of Desferrioxamine B. *J. Incl*. Phenom. Mol. Recognit. Chem..

[134] Kalinowski B.E., Oskarsson A., Albinsson Y., Arlinger J., Ödegaard-Jensen A., Andlid T., Pedersen K. (2004). Microbial leaching of uranium and other trace elements from shale mine tailings at Ranstad. Geoderma.

[135] Schalk I.J., Hannauer M., Braud A. (2011). New roles for bacterial siderophores in metal transport and tolerance. Environ. Microbiol..

[136] Ahmed E., Holmström S.J.M. (2014). Siderophores in environmental research: Roles and applications. Microb. Biotechnol..

[137] Barbeau K., Zhang G., Live D.H., Butler A. (2002). Petrobactin, a Photoreactive Siderophore Produced by the Oil-Degrading Marine Bacterium *Marinobacter hydrocarbonoclasticus*. J. Am. Chem. Soc..

[138] Raju M., Srivastava S., Nair R.R., Raval I.H., Haldar S., Chatterjee P.B. (2017). Siderophore coated magnetic iron nanoparticles: Rational designing of water soluble nanobiosensor for visualizing Al^3+^ in live organism. Biosens. Bioelectron..

[139] Su B.-L., Moniotte N., Nivarlet N., Tian G., Desmet J. (2010). Design and synthesis of fluorescence-based siderophore sensor molecules for Fe III ion determination. Pure Appl. Chem..

[140] Yin K., Wu Y., Wang S., Chen L. (2016). A sensitive fluorescent biosensor for the detection of copper ion inspired by biological recognition element pyoverdine. Sens. Actuators B Chem..

[141] Yin K., Zhang W., Chen L. (2014). Pyoverdine secreted by *Pseudomonas aeruginosa* as a biological recognition element for the fluorescent detection of furazolidone. Biosens. Bioelectron..

[142] Hu J., Ghosh M., Miller M.J., Bohn P.W. (2019). Whole-cell biosensing by siderophore-based molecular recognition and localized surface plasmon resonance. Anal. Methods.

[143] Doorneweerd D.D., Henne W.A., Reifenberger R.G., Low P.S. (2010). Selective Capture and Identification of Pathogenic Bacteria Using an Immobilized Siderophore. Langmuir.

[144] Kim Y., Lyvers D.P., Wei A., Reifenberger R.G., Low P.S. (2012). Label-free detection of a bacterial pathogen using an immobilized siderophore, deferoxamine. Lab Chip.

